# Systemic steroidophobia scale for pediatric rheumatology (SSS-PR): development and validation of a novel psychometric tool measuring clinician steroidophobia

**DOI:** 10.1186/s12969-026-01200-z

**Published:** 2026-03-26

**Authors:** Sema Nur Taşkın, Sevil Aydoğan Gedik, Emrah Atay, Zeynep Kasap Acungil, Nuray Aktay Ayaz

**Affiliations:** 1 Division of Pediatric Rheumatology, Department of Pediatrics, Eskisehir City Hospital, Eskisehir, Türkiye; 2 Department of Public Health, Eskişehir Provincial Directorate of Health, Eskişehir, Türkiye; 3 Department of Physiotherapy and Rehabilitation, Faculty of Health Science, Tokat Gaziosmanpasa University, Tokat, Türkiye; 4 Division of Pediatric Rheumatology, Department of Pediatrics, İstanbul Faculty of Medicine, İstanbul University, İstanbul, Türkiye

**Keywords:** Systemic corticosteroids, Steroidophobia, Scale development, Validity and reliability, Pediatric rheumatology, Clinician attitudes, Medical education

## Abstract

**Background:**

Systemic corticosteroids are cornerstone therapies in pediatric inflammatory diseases. Despite their clinical importance, both parents and clinicians may harbor substantial concerns regarding steroid-related adverse effects. While “steroidophobia” has been extensively studied in the context of topical corticosteroids, no validated instrument exists to assess clinician-based attitudes toward systemic corticosteroid use. This study aimed to develop and validate novel psychometric tools to measure systemic steroidophobia among clinicians.

**Methods:**

A methodological, multicenter study was conducted between June and October 2025 among pediatric rheumatology specialists and fellows. Two draft instruments were developed: the Oral Corticosteroid Phobia Scale (21 items) and the Intravenous Corticosteroid Phobia Scale (22 items). Face and content validity were evaluated through expert review. Item analysis, exploratory and confirmatory factor analyses, internal consistency (Cronbach’s alpha), and test–retest reliability were performed.

**Results:**

A total of 121 clinicians participated. After item reduction, the oral scale comprised 15 items and the intravenous scale 16 items, each demonstrating a two-factor structure. The Oral Corticosteroid Phobia Scale explained 63.5% of total variance (Cronbach’s alpha = 0.908), and the Intravenous Corticosteroid Phobia Scale explained 66.4% (Cronbach’s alpha = 0.935). Test–retest reliability was excellent (ICC = 0.902 and 0.930, respectively). Model fit indices indicated good construct validity. Lower phobia scores were observed among clinicians with longer pediatric rheumatology experience.

**Conclusions:**

The developed scales demonstrated acceptable validity and reliability for assessing clinician-based systemic steroidophobia. These tools may facilitate a more systematic and objective identification of attitudinal barriers toward corticosteroid use and may serve as formative educational instruments in residency training and continuing medical education. Further validation across different pediatric subspecialties and countries is warranted.

**Supplementary Information:**

The online version contains supplementary material available at 10.1186/s12969-026-01200-z.

## Introduction

Glucocorticoid (steroid) agents are fundamental components in the treatment of rheumatologic diseases, playing a crucial role in controlling inflammation and maintaining immune regulation. However, the use of steroids in pediatric patients requires particular caution due to their potential adverse effects, most notably on bone growth, bone mineral density, adrenal suppression, metabolic disturbances, and increased risk of infections [1].

These risks become more pronounced particularly with systemic and long-term steroid therapy [2]. In contrast, although data on short-term steroid use are generally limited, meta-analyses indicate that major adverse effects such as hyperglycemia, sleep disturbances, and gastrointestinal bleeding may still occur [3].

In pediatric rheumatology practice, the decision to initiate steroid therapy is influenced not only by disease activity but also by multiple factors such as the patient’s age, duration of treatment, dose selection, concomitant medications, and individual patient characteristics. Moreover, variations in clinicians’ attitudes toward steroid use, their level of knowledge, and factors such as “steroid fear” (steroid phobia) may lead to discrepancies in clinical decision-making.

In contemporary medical doctrine, steroid therapy occupies a highly significant position in terms of both its frequency of use and the number of specialties in which it is preferred. Although specific treatment regimens are applied based on defined clinical scenarios, objective tools that systematically assess clinicians’ concerns and perspectives regarding different doses, routes of administration, and potential adverse effects have not been specifically developed for systemic corticosteroid use in pediatric rheumatology. For instance, the Topical Corticosteroid Phobia Scale (TOPICOP), developed to evaluate concerns related to topical steroids in dermatology practice, is an important instrument for assessing clinician and patient attitudes; however, it focuses exclusively on topical formulations and therefore is not applicable to most pediatric rheumatologic indications [4]. On the other hand, approaches in the literature that address the systemic use of steroids—namely oral and intravenous formulations—remain markedly limited. This gap is particularly evident in pediatric rheumatology, where, to the best of our knowledge, no validated instrument specifically designed to assess such concerns has been developed. In this context, the aim of our study was to develop valid and reliable measurement tools to evaluate systemic steroidophobia in pediatric rheumatology practice and to contribute to a more systematic and objective assessment of clinicians’ attitudes toward systemic corticosteroid use.

## Materials and methods

### Study design and study population

This study was a methodological study conducted between June 2025 and October 2025 among pediatric rheumatology specialists and subspecialty fellows.

One commonly accepted approach in scale development studies is to determine the sample size based on the number of items included in the scale. It is generally recommended that the sample size be between 5 and 20 times the number of items [5]. In the present study, a ratio of five participants per item was adopted in accordance with the recommended range. This threshold is generally considered acceptable for preliminary factor analysis in methodological validation studies. Since the “Oral Corticosteroid Phobia Scale” consisted of 21 items and the “Intravenous Corticosteroid Phobia Scale” consisted of 22 items, the minimum required sample size was calculated accordingly, and the larger value—110 participants—was set as the target minimum sample size.

A convenience sampling strategy, one of the non-probability sampling methods, was employed to recruit participants. The target population consisted of pediatric rheumatology specialists and pediatric rheumatology subspecialty fellows actively practicing in Türkiye. The survey link, developed using Google Forms, was disseminated to a national pediatric rheumatology professional communication group comprising 207 members. To ensure comprehensive distribution, the invitation was announced through both e-mail and WhatsApp communication channels.

Among the respondents, 11 participants completed the questionnaire via the e-mail-distributed link, while the remaining participants accessed the survey through the link shared on WhatsApp. After the predefined minimum sample size was reached, data collection continued until no further responses were received. Ultimately, a total of 121 individuals were included in the study.

Participation was entirely voluntary. As the survey link was distributed through open professional communication networks, the exact number of clinicians who viewed the invitation could not be ascertained; therefore, a precise response rate could not be calculated. All responses were collected and analyzed anonymously.

This study was approved by the Scientific Research Ethics Committee of Eskişehir City Hospital on 12/06/2025 with decision number ESH/BAEK-2025/169. The research was conducted in accordance with the ethical principles of the Declaration of Helsinki. Responses to the scale items were used solely for scientific purposes; no identifying information was collected from participants, and all data were analyzed anonymously.

### Data collection instruments

The first section of the questionnaire includes items assessing certain sociodemographic characteristics and professional attributes. The second section contains the designed items belonging to the “*Oral Corticosteroid Phobia Scale*” and the “*Intravenous Corticosteroid Phobia Scale*.”

To identify pediatric rheumatology specialists’ perspectives on oral corticosteroid use, a 21-item draft scale—the “*Oral Corticosteroid Phobia Scale*”—was developed following a structured review of the literature on corticosteroid-related adverse effects and previously validated steroid phobia instruments, including the TOPICOP scale [2, 4, 6]. Similarly, to assess their perspectives on intravenous corticosteroid use, a 22-item draft scale—the “*Intravenous Corticosteroid Phobia Scale*”—was created based on the same conceptual framework. Both scales employ a five-point Likert format with response options of “strongly disagree, disagree, neutral, agree, and strongly agree” [7, 8]. Each item is scored from 1 to 5, and higher total scores indicate a greater level of corticosteroid phobia in either oral or intravenous administration, depending on the scale type.

### Assessment of face validity

To evaluate the face validity of the “*Oral Corticosteroid Phobia Scale*” and the “*Intravenous Corticosteroid Phobia Scale*,*”* the opinions of 21 experts in the field of pediatric rheumatology were obtained. The experts assessed whether the scale items appeared appropriate for their intended purpose, whether they conceptually reflected the construct they were designed to measure, whether the items were relevant to the intended content, whether the title and the content of the scale were compatible, and whether the items were clear and understandable. The experts involved in the face validity assessment were pediatric rheumatology specialists working at tertiary referral and university hospitals across Türkiye. They were identified through professional networks and invited individually via e-mail and WhatsApp communication channels. Participation was voluntary, and no financial compensation was provided.

All experts responded with “*agree*” or “*strongly agree*” to the statements “*the title and content of the scale are compatible with each other*” and “*the items are clear and understandable*.” Twenty of the experts marked “*agree*” or “*strongly agree*” for the statements “*the scale items conceptually reflect the construct they are intended to measure*,” “*the content of the scale appears appropriate for its purpose*,” and “*the items included in the scale are relevant to the intended topic*,*”* whereas only one expert selected “*neutral*” for these three statements. Based on the expert evaluations, it was concluded that both scales demonstrated adequate face validity.

### Content validity ratio and content validity index

Expert opinion was sought to evaluate content validity. The 22-item draft scale for the intravenous form was presented to 15 experts holding pediatric subspecialties from different fields. These experts were pediatric subspecialists working at tertiary referral and university hospitals across Türkiye, actively involved in the clinical use of systemic corticosteroids and experienced in managing corticosteroid-related adverse effects. The panel included pediatric rheumatologists as well as specialists from a broad range of pediatric subspecialties involved in the clinical management of conditions requiring systemic corticosteroid therapy. They were selected based on their clinical experience and academic background and were invited individually through professional networks via e-mail and WhatsApp communication channels. Participation was voluntary, and no financial compensation was provided.

Experts evaluated each item of the scale using three response options: “not appropriate,” “needs revision,” and “appropriate.” The Content Validity Ratio (CVR) for each item was calculated in accordance with the method proposed by Lawshe, by dividing the number of experts who rated the item as “appropriate” by half of the total number of experts and subtracting 1 from the resulting value [8]. For a panel of 15 experts, a minimum CVR value of 0.49 is recommended according to Lawshe’s critical values table; items with lower values should be removed from the scale [8]. In the present study, the lowest calculated CVR was 0.86; therefore, no items were eliminated. Subsequently, the Content Validity Index (CVI) for the overall scale was calculated by summing the CVR values of all items and dividing by the total number of items. A CVI value greater than 0.67 is considered acceptable [5]. The calculated CVI was 0.98, indicating satisfactory content validity of the scale.

For the oral form, the 21-item draft scale was similarly presented to the same 15 experts. Based on the evaluations performed in the same manner as for the intravenous form, the CVR and CVI values were calculated. All experts rated every item as “*the item is appropriate.”* Therefore, all calculated CVR values were 1, and no items were removed from the scale. The CVI of the scale was also found to be 1, indicating that content validity was achieved.

### Item analysis and assessment of internal consistency

Item analysis was conducted to examine the contribution of each item to the scale. In this context, item–total correlation coefficients were calculated. Attention was paid to ensuring that the item–total correlations were positive and greater than 0.20 [7]. Additionally, the Cronbach’s alpha coefficient of the scale was calculated, and changes in this reliability coefficient were examined when items were deleted. As another control step, within the framework of the item discrimination approach, the “*item discrimination power index*” was evaluated by comparing the item means of the lower and upper 27% groups in accordance with Kelley’s method [9]. Since the groups were found to be non-normally distributed, the Mann–Whitney U test was used for the comparisons.

### Exploratory and confirmatory factor analysis

Construct validity was evaluated using factor analysis. Both exploratory and confirmatory factor analyses were performed. The exploratory factor analysis was conducted using the SPSS statistical package program (v.15). In determining the number of factors, components with eigenvalues greater than 1 and those explaining more than 5% of the additional variance were considered [5, 10]. In addition, the scree plot was examined. As part of the advanced assessment of factor analysis, a factor rotation procedure was performed using the varimax rotation method. For factor loadings, a minimum value of 0.30 was accepted [5].

For the confirmatory factor analysis, outliers in the dataset were first removed using the Shiny application. Confirmatory factor analysis was then performed in the Jamovi 2.7.12 program [11]. Since multivariate normality was not achieved, a robust estimation method was used.

### Test–retest application

A test–retest procedure was conducted to evaluate stability, which is a component of reliability. The questionnaire was administered to 25 pediatric rheumatology specialists, and the same questionnaire was re-administered two weeks later. Since the distribution of the scores obtained from the scales in both administrations met the assumption of normality, the Pearson correlation coefficient and the Intraclass Correlation Coefficient (ICC) were calculated to assess the relationship between the pre-test and post-test scores.

A significance level of *p* < 0.05 was accepted as the threshold for all statistical analyses.

## Results

### Descriptive characteristics

A total of 121 pediatric rheumatology clinicians were included in the study. Their baseline characteristics, including age distribution, are summarized in Table 1.

**Table 1:** Demographic and professional characteristics of participants (*n* = 121)

Characteristics	Value
**Age (years)**,** mean ± SD**	40.5 ± 7.7
**Years in medical practice**,** mean ± SD**	16.4 ± 7.9
**Years in pediatric rheumatology practice**,** mean ± SD**	6.6 ± 6.8
**Sex**,** n (%)**	
Female	94 (77.7)
Male	27 (22.3)
**Age group**,** n (%)**	
≤45 years	97 (80.2)
≥46 years	24 (19.8)
**Years in medical practice**,** n (%)**	
≤10 years	29 (24.0)
11–20 years	67 (55.4)
≥21 years	25 (20.6)
**Years in pediatric rheumatology practice**,** n (%)**	
≤10 years	106 (87.6)
11–20 years	8 (6.6)
≥21 years	7 (5.8)
**Academic title**,** n (%)**	
Pediatric Rheumatology Fellow	43 (35.6)
Specialist (MD)	32 (26.4)
Assistant Professor	1 (0.8)
Associate Professor	31 (25.6)
Professor	14 (11.6)
**Institution type**,** n (%)**	
Public university hospital	77 (63.6)
Non-university public hospital	38 (31.4)
Foundation university hospital	2 (1.7)
Private hospital	3 (2.5)
Private practice	1 (0.8)
**Weekly frequency of encountering patients requiring corticosteroid therapy**,** n (%)**	
1–2 patients/week	20 (16.5)
3–5 patients/week	33 (27.3)
6–10 patients/week	31 (25.6)
>10 patients/week	37 (30.6)

### Item analysis and internal consistency analyses

For the “*Oral Corticosteroid Phobia Scale”*, three items with item–total correlation coefficients below 0.20 were removed from the scale, and the excluded items were as follows [7]:

- *I discontinue oral corticosteroid therapy as early as possible.*
- *Before initiating long-term oral corticosteroid treatment (longer than two weeks)*,* I inform the family that stopping the medication without consulting a physician may lead to serious adverse effects and that the medication should be tapered gradually (with a tapering schedule).*
- *While the patient is using oral corticosteroid therapy*,* I inform the family about additional management measures such as salt-restricted diet*,* blood pressure and blood glucose monitoring*,* and the importance of vitamin D supplementation.*
- *Subsequently*,* calculations were repeated with the remaining 18 items. One additional item with an item–total correlation coefficient below 0.20 was removed from the scale*,* and the excluded item was as follows*:
- *I inform the family about the potential side effects before initiating oral corticosteroid therapy.*

In the repeated analysis conducted with the remaining 17 items, the Cronbach’s alpha reliability coefficient was found to be 0.902. The Cronbach’s alpha values calculated when any single item was deleted ranged from 0.887 to 0.905. The item–total correlation coefficients varied between 0.243 and 0.707.

For the “*Intravenous Corticosteroid Phobia Scale*,” four items with item–total correlation coefficients below 0.20 were removed from the scale, and the excluded items were as follows [7]:

- *I discontinue intravenous corticosteroid therapy as early as possible.*
- *I inform the family about the potential side effects before initiating intravenous corticosteroid therapy.*
- *Before initiating long-term intravenous corticosteroid therapy (longer than 2 weeks)*,* I inform the family that abruptly discontinuing the medication without consulting the physician may cause serious adverse effects*,* and that the dose should be tapered according to a planned tapering schedule.*
- *During intravenous corticosteroid therapy*,* I inform the family about adjunctive measures such as a low-salt diet*,* monitoring blood pressure and blood glucose levels*,* and the importance of vitamin D supplementation.*

With the remaining 18 items, the Cronbach’s alpha value was calculated as 0.930. The item–total correlation coefficients ranged between 0.222 and 0.730, and the Cronbach’s alpha values obtained when any item was deleted varied between 0.920 and 0.933.

### Comparison of scores between the lower and upper 27% groups

Following the item analysis, the scores obtained from the 17-item “*Oral Corticosteroid Phobia Scale*” were ranked from highest to lowest, and the upper and lower 27% groups were formed. Each group consisted of 33 participants. When the total scale scores and individual item scores were compared between these groups, the scores of the upper 27% group (total scale score: mean 69.8 ± 4.4, median 69.0) were found to be significantly higher than those of the lower 27% group (total scale score: mean 41.6 ± 7.4, median 44.0) (*p* < 0.001 for each comparison).

The same procedure was repeated for the “*Intravenous Corticosteroid Phobia Scale*”, which had been reduced to 18 items. The scores of the upper 27% group (total scale score: mean 77.6 ± 6.1, median 78.0) were found to be higher than those of the lower 27% group (total scale score: mean 42.8 ± 7.7, median 43.0), both in terms of total scale scores and individual item scores (*p* < 0.001 for each comparison).

All items in both scales demonstrated adequate discriminatory power, and therefore no items were eliminated at this stage.

### Exploratory factor analysis

Following item analysis, the “*Oral Corticosteroid Phobia Scale*” was reduced to 17 items. The suitability of the data for factor analysis was assessed, yielding a Kaiser–Meyer–Olkin (KMO) measure of sampling adequacy of 0.889, and Bartlett’s test of sphericity was statistically significant (*p* < 0.001). Examination of the correlation matrix revealed no evidence of singularity or multicollinearity. These findings indicated that the dataset was appropriate for factor analysis.

During the exploratory factor analysis, the item “Before prescribing oral corticosteroid therapy, I consider all other treatment options and view corticosteroids as a last resort” was found to exhibit cross-loadings on two factors and was therefore excluded from the scale, after which the analysis was repeated. In addition, another item—“Before initiating oral corticosteroid therapy, I re-review the literature regarding its side effects”—was observed to load independently onto a separate factor. As it is recommended that each factor should comprise at least three items prior to conducting confirmatory factor analysis, this item was also removed, and the exploratory factor analysis was repeated before proceeding to the confirmatory factor analysis [12, 13]:

Following these refinements, the “*Oral Corticosteroid Phobia Scale*”, reduced to 15 items, was found to comprise two subdimensions, explaining 63.5% of the total variance. Factor loadings ranged from 0.556 to 0.894. The Cronbach’s alpha coefficient for the final 15-item version was recalculated and yielded a value of 0.908, indicating excellent internal consistency. Item–total correlation coefficients ranged from 0.374 to 0.722 (Table 2).

**Table 2:** Factor structure of the oral corticosteroid phobia scale

Oral Corticosteroid Phobia Scale		Factor Loading*		Item–Total Correlation Coefficient	Cronbach’s Alpha if Item Deleted
Factor 1 Initial Eigenvalue: 6.864 Explained Variance: 45.7% Cronbach’s Alpha: 0.930	Item 1	0.848		0.703	0.897
	Item 2	0.825		0.722	0.895
	Item 3	0.811		0.711	0.896
	Item 4	0.807		0.710	0.896
	Item 5	0.790		0.705	0.896
	Item 6	0.789		0.693	0.897
	Item 7	0.776		0.669	0.898
	Item 8	0.740		0.575	0.901
	Item 9	0.708		0.700	0.896
	Item10	0.693		0.660	0.898
	Item 11	0.556		0.480	0.904
Factor 2 Initial Eigenvalue: 2.66 Explained Variance: 17.8% Cronbach’s Alpha: 0.880	Item 12		0.894	0.398	0.908
	Item 13		0.867	0.374	0.909
	Item 14		0.828	0.413	0.906
	Item 15		0.804	0.468	0.905
Total Explained Variance: 63.5% Overall Cronbach’s Alpha: 0.908					

*Factor structure and factor loadings obtained using varimax rotation

Following item analysis, the “*Intravenous Corticosteroid Phobia Scale”* was reduced to 18 items. The suitability of the data for factor analysis was evaluated, yielding a KMO value of 0.915, and Bartlett’s test of sphericity was statistically significant (p < 0.001). Examination of the correlation matrix indicated no evidence of singularity or multicollinearity, confirming that the dataset was appropriate for factor analysis.

Exploratory factor analysis revealed a three-factor structure. However, the third factor was represented by only two items, which were as follows:

- Before administering intravenous corticosteroid therapy, I consider all alternative treatment options and regard corticosteroids as a last resort.
- Before initiating intravenous corticosteroid therapy, I re-review the literature regarding its side effects.

As it is recommended that each factor should comprise at least three items in confirmatory factor analysis, these two items were removed from the scale prior to proceeding with confirmatory factor analysis, and the exploratory factor analysis was subsequently repeated [12, 13]:

Following these revisions, the “*Intravenous Corticosteroid Phobia Scale*”, reduced to 16 items, was found to consist of two subdimensions, explaining 66.4% of the total variance. Factor loadings ranged from 0.654 to 0.865. The Cronbach’s alpha coefficient for the final 16-item version was recalculated and yielded a value of 0.935, indicating excellent internal consistency. Item–total correlation coefficients ranged from 0.573 to 0.720 (Table 3).

**Table 3:** Factor structure of the intravenous corticosteroid phobia scale

Intravenous Corticosteroid Phobia Scale		Factor Loading*		Item–Total Correlation Coefficient	Cronbach’s Alpha if Item Deleted
Factor 1 Initial Eigenvalue: 8.154 Explained Variance: 50.9% Cronbach’s Alpha: 0.933	Item 1	0.865		0.706	0.928
	Item 2	0.833		0.697	0.928
	Item 3	0.792		0.720	0.928
	Item 4	0.784		0.655	0.929
	Item 5	0.755		0.672	0.929
	Item 6	0.741		0.671	0.929
	Item 7	0.710		0.718	0.928
	Item 8	0.691		0.719	0.928
	Item 9	0.686		0.573	0.931
	Item 10	0.674		0.674	0.929
	Item 11	0.654		0.596	0.931
Factor 2 *Initial Eigenvalue*: 2.475 *Explained Variance*: 15.4% *Cronbach’s Alpha*: 0.931	Item12		0.907	0.673	0.929
	Item13		0.867	0.620	0.930
	Item 14		0.864	0.660	0.929
	Item 15		0.861	0.609	0.931
	Item 16		0.783	0.658	0.929
Total Explained Variance: 66.4% Overall Cronbach’s Alpha: 0.935					

* Factor structure and factor loadings obtained using varimax rotation

### Confirmatory factor analysis

**Fig. 1:**
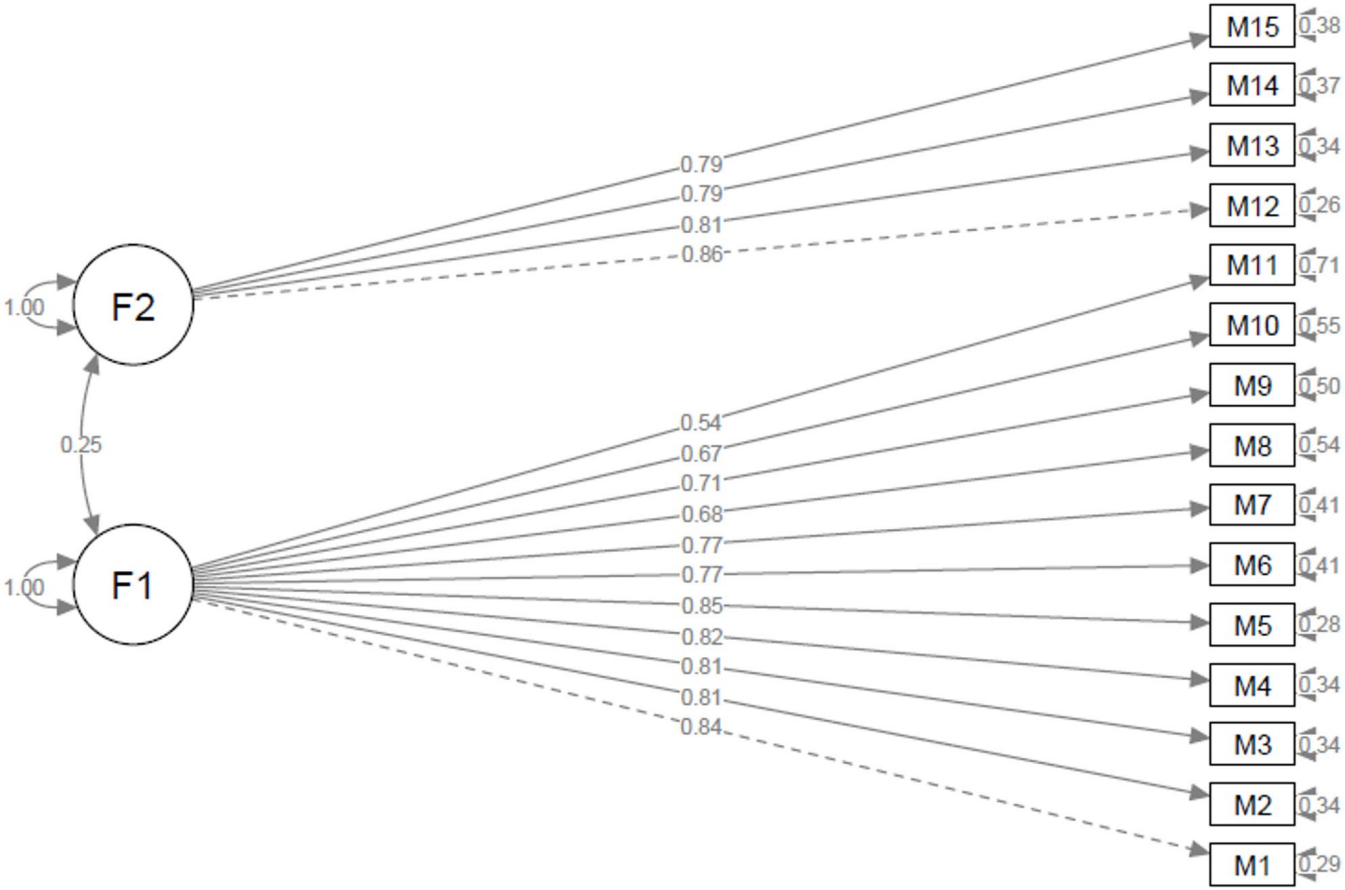
Path diagram of the oral corticosteroid phobia scale

**Fig. 2:**
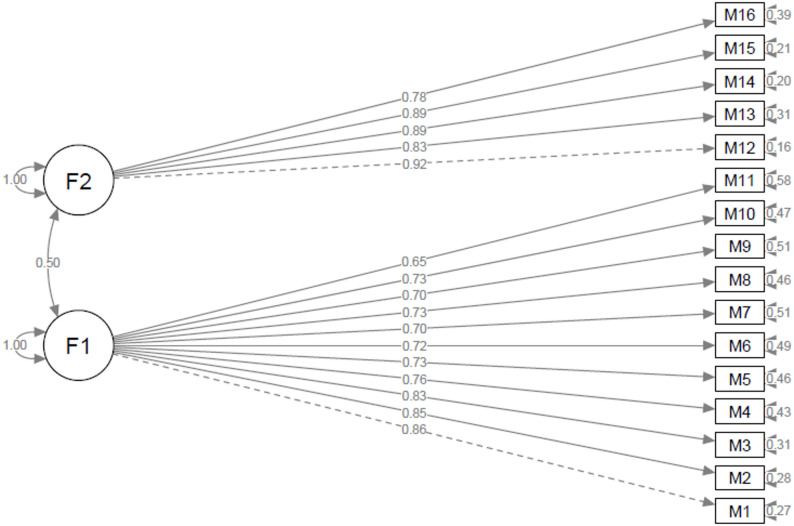
Path diagram of the intravenous corticosteroid phobia scale

Using the Shiny application, three outliers were identified and removed for the “*Oral Corticosteroid Phobia Scale*”, and the confirmatory factor analysis was conducted on 118 participants. For the “*Intravenous Corticosteroid Phobia Scale*”, seven outliers were excluded, and the analysis was performed on 114 participants. For both scales, most goodness-of-fit indices were within acceptable ranges or were close to these thresholds (Table 4) [12, 14]. Path diagrams illustrating the model structure and factor loadings for each scale are presented in Figs. 1 and 2.

**Table 4:** Model fit indices for the oral and intravenous corticosteroid phobia scales

Fit Index	Acceptable Threshold	Oral Corticosteroid Phobia Scale		Intravenous Corticosteroid Phobia Scale	
		Value	Interpretation	Value	Interpretation
χ²/df	< 5	167/89 = 1.87	Excellent fit	208/103 = 2.01	Excellent fit
RMSEA	< 0.08	0.084	Slightly above acceptable threshold	0.088	Slightly above acceptable threshold
SRMR	< 0.08	0.058	Good fit	0.056	Good fit
CFI	> 0.90	0.934	Good fit	0.931	Good fit
TLI	> 0.80	0.922	Good fit	0.920	Good fit

### Comparison of scores between the lower and upper 27% groups

Following factor analysis, the “*Oral Corticosteroid Phobia Scale*” was reduced to 15 items. Total scores obtained from the scale were ranked from highest to lowest, and the upper and lower 27% groups were identified. Each group consisted of 33 participants. Comparisons of total scale scores and individual item scores between these groups demonstrated that the upper 27% group had significantly higher scores (mean total score 62.9 ± 4.3, median 61.0) than the lower 27% group (mean total score 37.4 ± 7.2, median 39.0). The differences were statistically significant for both the total scale score and all individual items (*p* < 0.001 for each comparison).

The same procedure was applied to the “*Intravenous Corticosteroid Phobia Scale*”, which was reduced to 16 items. Similarly, the upper 27% group had significantly higher scores (mean total score 70.1 ± 5.8, median 70.0) than the lower 27% group (mean total score 38.3 ± 7.3, median 39.0), both in terms of the total scale score and individual item scores (*p* < 0.001 for each comparison).

### Test–retest reliability

A total of 25 pediatric rheumatology specialists participated in the test–retest analysis. For the 15-item “*Oral Corticosteroid Phobia Scale*”, baseline (test) scores ranged from 28 to 70, with a mean of 48.8 ± 10.4, while baseline scores for the 16-item “*Intravenous Corticosteroid Phobia Scale*” ranged from 32 to 80, with a mean of 52.8 ± 12.4. At the retest assessment, scores on the oral form ranged from 32 to 71 (mean 49.3 ± 10.5), and scores on the intravenous form ranged from 32 to 76 (mean 51.8 ± 13.2).

For the oral form, a strong positive correlation was observed between baseline and retest scores (*r* = 0.842, *p* < 0.001). The intraclass correlation coefficient (ICC) between the two measurements was 0.902 (95% CI: 0.834–0.950, *p* < 0.001).

Similarly, for the intravenous form, a strong positive correlation was found between baseline and retest scores (*r* = 0.903, *p* < 0.001), with an ICC of 0.930 (95% CI: 0.882–0.964, *p* < 0.001). These findings indicate that both the oral and intravenous forms demonstrate excellent test–retest reliability, remaining stable over time.

### Distribution of scores obtained from the final scales

Scores obtained from the final validated 15-item “*Oral Corticosteroid Phobia Scale*” ranged from 15 to 75, with a mean of 50.9 ± 10.7. Scores derived from the final 16-item “*Intravenous Corticosteroid Phobia Scale*” ranged from 20 to 80, with a mean of 54.7 ± 13.0.

Physicians with more than 21 years of experience in pediatric rheumatology had lower scores on the “*Oral Corticosteroid Phobia Scale*”. Similarly, these physicians also demonstrated lower scores on the “*Intravenous Corticosteroid Phobia Scale*” compared with physicians who had less than 10 years of experience in the field of pediatric rheumatology (Table 5).

**Table 5:** Distribution of oral and intravenous corticosteroid phobia scale scores according to sociodemographic characteristics

Characteristics	Oral Corticosteroid Phobia Scale		Intravenous Corticosteroid Phobia Scale	
	Median (Min-Max)	*p*	Median (Min-Max)	*p*
Sex		0.322		0.320
Female	52 (15–75)		55 (20–80)	
Male	50 (25–68)		53 (26–75)	
Age		0.640		0.460
≤45	51 (25–75)		55 (26–80)	
≥46	53 (15–70)		54 (20–80)	
Years in Medical Practice		0.572		0.368
≤10 years	50 (32–69)		55 (33–70)	
11–20 years	52 (25–75)		54 (26–80)	
≥21 years	52 (15–70)		53 (20–78)	
Years in Pediatric Rheumatology Practice		**0.021**		**0.018**
≤10 years	52 (25–75)		55 (26–80)*	
11–20 years	57 (45–69)		57 (45–73)	
≥21 years	37 (15–61)*		33(20–58)*	
Academic Title		0.507		0.067
Pediatric Rheumatology Fellow	51(28–70)		57 (32–77)	
Specialist (MD)	52 (37–75)		54 (36–80)	
Assistant Professor	57 (57–57)		75 (75–75)	
Associate Professor	53 (25–69)		53 (26–78)	
Professor	45.5 (15–69)		48 (20–73)	
Institution Type		0.150		0.167
Non-university public hospital	49.5 (25–75)		54 (26–77)	
Public university hospital	51 (15–70)		55 (20–80)	
Foundation university hospital	51 (45–57)		60 (45–75)	
Private hospital	61 (58–64)		65 (58–80)	
Private practice	69 (69–69)		78 (78–78)	
Weekly Frequency of Encountering Patients Requiring Corticosteroid Therapy		0.051		0.116
1–2 patients/week	58 (31–69)		56.5 (32–80)	
3–5 patients/week	48 (25–60)		51 (26–70)	
6–10 patients/week	52 (28–67)		55 (26–77)	
>10 patients per week	53 (15–75)		57 (20–77)	
Total	52 (15–75)		55 (20–80)	

*Group responsible for the observed difference

Examination of responses to the “*Oral Corticosteroid Phobia Scale*” indicated that, for the majority of items, participants most frequently selected “partially agree” or “agree.” In contrast, responses of “strongly disagree” or “disagree” were most commonly reported for the following items: “I am concerned about the genitourinary side effects of oral corticosteroids,” “I am reluctant to prescribe oral corticosteroids at doses exceeding 1 mg/kg/day,” “I am reluctant to prescribe oral corticosteroids to children younger than two years of age,” “I am reluctant to prescribe oral corticosteroids even at doses up to 1 mg/kg/day,” and “Despite being aware of steroid-related side effects, I am reluctant to prescribe oral corticosteroids.” The distribution of responses to items of the “*Oral Corticosteroid Phobia Scale*” among study participants is presented in Table 6.

**Table 6:** Distribution of responses to items of the oral corticosteroid phobia scale

Oral Corticosteroid Phobia Scale	*n* (%)				
	Strongly disagree	Disagree	Neutral	Partially agree	Strongly agree
1. I am concerned about the musculoskeletal side effects of oral corticosteroids.	2 (1.7)	14 (11.6)	14 (11.6)	58 (47.9)	33 (27.3)
2. I am concerned about the neuropsychiatric side effects of oral corticosteroids.	4 (3.3)	21 (17.4)	16 (13.2)	48 (39.7)	32 (26.4)
3. I am concerned about the metabolic–endocrinological side effects of oral corticosteroids.	5 (4.1)	8 (6.6)	9 (7.4)	56 (46.3)	43 (35.5)
4. I am concerned about the ophthalmological side effects of oral corticosteroids.	2 (1.7)	11 (9.1)	11 (9.1)	47 (38.8)	50 (41.3)
5. I am concerned about the immunological side effects of oral corticosteroids.	3 (2.5)	12 (9.9)	8 (6.6)	49 (40.5)	49 (40.5)
6. I am concerned about the cardiovascular side effects of oral corticosteroids.	8 (6.6)	22 (18.2)	19 (15.7)	55 (45.5)	17 (14.0)
7. I am concerned that oral corticosteroids may cause growth retardation in children.	3 (2.5)	7 (5.8)	11 (9.1)	47 (38.8)	53 (43.8)
8. I am concerned about the dermatological side effects of oral corticosteroids.	5 (4.1)	28 (23.1)	12 (9.9)	56 (46.3)	20 (16.5)
9. I am concerned about the hematological side effects of oral corticosteroids.	5 (4.1)	30 (24.8)	7 (5.8)	54 (44.6)	25 (20.7)
10. I am concerned about the genitourinary side effects of oral corticosteroids.	13 (10.7)	43 (35.5)	23 (19.0)	32 (26.4)	10 (8.3)
11. I am concerned about the gastrointestinal side effects of oral corticosteroids.	1 (0.8)	9 (7.4)	9 (7.4)	52 (43.0)	50 (41.3)
12. I am reluctant to prescribe oral corticosteroids at doses exceeding 1 mg/kg/day.	18 (14.9)	54 (44.6)	11 (9.1)	30 (24.8)	8 (6.6)
13. I am reluctant to prescribe oral corticosteroids to children younger than two years of age.	21 (17.4)	50 (41.3)	13 (10.7)	31 (25.6)	6 (5.0)
14. I am reluctant to prescribe oral corticosteroids even at doses up to 1 mg/kg/day.	41 (33.9)	57 (47.1)	8 (6.6)	14 (11.6)	1 (0.8)
15. Despite being aware of steroid-related side effects, I am reluctant to prescribe oral corticosteroids.	24 (19.8)	62 (51.2)	11 (9.1)	21 (17.4)	3 (2.5)

An examination of responses to the “*Intravenous Corticosteroid Phobia Scale*” indicated that, for most items, participants predominantly selected “partially agree” or “agree.” In contrast, responses of “strongly disagree” or “disagree” were most frequently reported for the following items: “I am reluctant to prescribe intravenous corticosteroids even at doses up to 2 mg/kg/day,” “I am reluctant to prescribe intravenous corticosteroids to children younger than two years of age,” “I am reluctant to prescribe intravenous corticosteroids at doses between 2 and 10 mg/kg/day,” “I am reluctant to prescribe intravenous corticosteroids at doses between 10 and 30 mg/kg/day,” and “Despite being aware of steroid-related side effects, I am reluctant to prescribe corticosteroids via the intravenous route.” The distribution of responses to items of the “*Intravenous Corticosteroid Phobia Scale”* among study participants is presented in Table 7.

**Table 7:** Distribution of responses to items of the intravenous corticosteroid phobia scale

Intravenous Corticosteroid Phobia Scale	*n* (%)				
	Strongly disagree	Disagree	Neutral	Partially agree	Strongly agree
1. I am concerned about the musculoskeletal side effects of intravenously administered corticosteroids.	5 (4.1)	14 (11.6)	11 (9.1)	57 (47.1)	34 (28.1)
2. I am concerned about the neuropsychiatric side effects of intravenously administered corticosteroids.	5 (4.1)	16 (13.2)	11 (9.1)	54 (44.6)	35 (28.9)
3. I am concerned about the ophthalmological side effects of intravenously administered corticosteroids.	4 (3.3)	9 (7.4)	10 (8.3)	59 (48.8)	39 (32.2)
4. I am concerned about the cardiovascular side effects of intravenously administered corticosteroids.	2 (1.7)	19 (15.7)	14 (11.6)	54 (44.6)	32 (26.4)
5. I am concerned that intravenously administered corticosteroids may cause growth retardation in children.	3 (2.5)	12 (9.9)	10 (8.3)	52 (43.0)	44 (36.4)
6. I am concerned about the metabolic–endocrinological side effects of intravenously administered corticosteroids.	3 (2.5)	6 (5.0)	8 (6.6)	54 (44.6)	50 (41.3)
7. I am concerned about the genitourinary side effects of intravenously administered corticosteroids.	15 (12.4)	28 (23.1)	20 (16.5)	41 (33.9)	17 (14.0)
8. I am concerned about the hematological side effects of intravenously administered corticosteroids.	6 (5.0)	21 (17.4)	12 (9.9)	51 (42.1)	31 (25.6)
9. I am concerned about the dermatological side effects of intravenously administered corticosteroids.	4 (3.3)	32 (26.4)	13 (10.7)	49 (40.5)	23 (19.0)
10. I am concerned about the immunological side effects of intravenously administered corticosteroids.	3 (2.5)	10 (8.3)	7 (5.8)	46 (38.0)	55 (45.5)
11. I am concerned about the gastrointestinal side effects of intravenously administered corticosteroids.	3 (2.5)	8 (6.6)	15 (12.4)	51 (42.1)	44 (36.4)
12. I am reluctant to prescribe intravenous corticosteroids even at doses up to 2 mg/kg/day.	33 (27.3)	55 (45.5)	7 (5.8)	21 (17.4)	5 (4.1)
13. I am reluctant to prescribe intravenous corticosteroids to children younger than two years of age.	32 (26.4)	41 (33.9)	15 (12.4)	25 (20.7)	8 (6.6)
14. I am reluctant to prescribe intravenous corticosteroids at doses between 2 and 10 mg/kg/day.	26 (21.5)	49 (40.5)	8 (6.6)	25 (20.7)	13 (10.7)
15. I am reluctant to prescribe intravenous corticosteroids at doses between 10 and 30 mg/kg/day.	21 (17.4)	45 (37.2)	10 (8.3)	28 (23.1)	17 (14.0)
16. Despite being aware of steroid-related side effects, I am reluctant to prescribe corticosteroids via the intravenous route.	27 (22.3)	48 (39.7)	13 (10.7)	22 (18.2)	11 (9.1)

## Discussion

In pediatric rheumatology practice, systemic corticosteroids remain an important therapeutic option owing to their ability to rapidly suppress severe inflammation. Nevertheless, the potential for short- and long-term toxicities affecting metabolic, endocrine, dermatologic, cardiovascular, gastrointestinal, musculoskeletal, neuropsychiatric, ophthalmological, and immunological systems generates substantial concern among clinicians. In cases of prolonged or recurrent administration, a wide range of adverse effects has been described, including hyperglycemia, electrolyte disturbances, weight gain and Cushingoid appearance, hypertension, reduced bone mineral density, osteonecrosis, increased susceptibility to infections, neuropsychiatric manifestations, cataract formation, and the risk of adrenal insufficiency.

Particularly in children during critical growth periods, the suppression of linear growth velocity and the risk of delayed pubertal development represent some of the most sensitive clinical concerns. Consequently, many clinicians initiate high-dose corticosteroid therapy only when strictly necessary, aim to taper treatment as early as possible, and provide families with detailed counseling regarding potential complications [2, 15–17]. Collectively, these considerations suggest that clinicians’ attitudes toward corticosteroid use may be influenced by concerns specific to childhood growth and development.

The concept of “steroidophobia” has been defined primarily in the context of topical corticosteroid use, particularly in atopic dermatitis, and has been quantitatively assessed using the TOPICOP scale, which evaluates attitudes of both healthcare professionals and patients/parents toward topical corticosteroids [4, 18]. Previous studies have reported that a substantial proportion of parents perceive corticosteroids as dangerous, addictive, or permanently harmful medications, and that such perceptions are associated with reduced treatment adherence. However, existing research has largely been conducted in adult populations and within dermatology practice. To the best of our knowledge, no validated scale has been specifically developed to systematically assess clinician-oriented attitudes and concerns regarding systemic (oral or intravenous) corticosteroid use in pediatric rheumatology [4, 6, 18]. This study addresses this gap by providing the initial development and psychometric evaluation of instruments designed to assess pediatric rheumatologists’ reservations toward both intravenous and oral corticosteroid administration.

A recent systematic review and meta-analysis reported that short-term (≤ 14 days) systemic corticosteroid use in children and adolescents is most commonly associated with hyperglycemia and sleep disturbances, whereas the evidence for more severe complications, such as behavioral changes and severe gastrointestinal bleeding, remains limited and of low certainty. The authors further emphasized that serious adverse events necessitating early discontinuation of therapy are uncommon. Collectively, these findings suggest that concerns regarding severe complications during short treatment courses may not always be supported by high-certainty evidence [3]. In our study, clinicians reported higher levels of reluctance for items related to potential adverse effects, whereas dose-related items were associated with more limited concern. This observation suggests that clinician anxiety may be influenced more by the perceived clinical impact of adverse effects than by corticosteroid dosage itself.

Nevertheless, not only parents but also clinicians report substantial concerns regarding corticosteroid-related adverse effects. Such concerns have reinforced a tendency in pediatric rheumatology practice to administer systemic corticosteroids at the lowest effective dose for the shortest possible duration and to early adoption of steroid-sparing strategies [19]. Although this approach has the potential to reduce long-term toxicity, it may also carry the risk of insufficient initial suppression of severe inflammatory flares. Consequently, both parental pressure to avoid corticosteroid use and clinicians’ reluctance to initiate corticosteroid therapy may lead to delays in treatment initiation or premature dose tapering, particularly in conditions characterized by high inflammatory burden. The literature suggests that uncontrolled and prolonged inflammation can result in persistent multisystem effects, adversely affect growth, and increase the risk of irreversible tissue damage; therefore, such delays may have critical implications for long-term outcomes in pediatric patients [2, 6, 19]. This dual pressure—parental concern and clinician reluctance—has become increasingly visible in pediatric rheumatology, yet has not been systematically subjected to quantitative evaluation.

This study developed two distinct instruments—the “*Oral Corticosteroid Phobia Scale*” and the “*Intravenous Corticosteroid Phobia Scale”—*to assess fear and reluctance related to systemic corticosteroid use among pediatric rheumatology specialists, and presents their initial psychometric evaluation. The findings indicated that phobia scores were higher among clinicians with lower levels of professional experience. This observation may suggest that greater clinical experience is associated with increased treatment-related confidence and, consequently, lower levels of anxiety associated with corticosteroid use.

An additional consideration is the potential influence of professional experience on perceptions of systemic corticosteroid use. For many years, corticosteroids have represented one of the limited effective therapeutic options available in pediatric rheumatology. Clinicians trained in earlier therapeutic eras may therefore have developed greater familiarity and clinical confidence regarding systemic steroid use and its associated risks. In contrast, younger physicians and trainees practicing in a period characterized by the increasing availability of steroid-sparing therapies may adopt a more cautious approach toward corticosteroid administration.

These generational differences may not only reflect issues related to generalizability but may also indicate the scale’s capacity to capture meaningful variation in clinical perceptions and attitudes. In this context, the instrument may provide a structured framework for identifying beliefs and concerns that could influence treatment decisions across different stages of professional development.

The Cronbach’s alpha coefficient, which reflects internal consistency as a component of reliability, was 0.908 for the “*Oral Corticosteroid Phobia Scale*” and 0.935 for the “*Intravenous Corticosteroid Phobia Scale*”. A Cronbach’s alpha value between 0.80 and 1.00 indicates high reliability of a newly developed scale [5]. When considering the Cronbach’s alpha values for both the subscales and the total scales, these findings suggest that both instruments demonstrate a high level of internal consistency and reliability.

The item–total correlation coefficient represents the correlation between a given item and the sum of the remaining items in the scale. It is used to evaluate whether an individual item is consistent with the overall scale structure. An item–total correlation coefficient below 0.20 indicates poor item performance, and removal of such an item from the scale is generally recommended [7, 10]. In our study, items with low item–total correlation coefficients were excluded in accordance with this criterion. In the final versions of the scales, item–total correlation coefficients ranged from 0.374 to 0.722 for the “*Oral Corticosteroid Phobia Scale*” and from 0.573 to 0.720 for the “*Intravenous Corticosteroid Phobia Scale*”, indicating adequate item discrimination and acceptable internal consistency.

In the assessment of stability, which represents another component of reliability, test–retest analysis is employed, and high correlation coefficients and intraclass correlation coefficient (ICC) values between the two measurements are desirable. Values of 0.70 or higher are generally considered indicative of adequate temporal stability of a scale [5]. In our study, the correlation coefficient between test and retest administrations was 0.842, and the intraclass correlation coefficient (ICC) was 0.902 for the “*Oral Corticosteroid Phobia Scale*”. For the “*Intravenous Corticosteroid Phobia Scale*”, the correlation coefficient was 0.903, and the ICC was 0.930. These findings suggest that both scales demonstrate adequate temporal stability and remain relatively consistent over time across repeated measurements.

A factor loading represents the degree to which an item contributes to its corresponding subscale and to the overall construct of the scale. The literature reports various cutoff values for interpreting factor loadings. According to one commonly used classification, a factor loading of 0.71 (explaining 50% of the variance) is considered excellent, 0.63 (40% of the variance) very good, 0.55 (30% of the variance) good, 0.45 (20% of the variance) fair, and 0.32 (10% of the variance) poor [12]. According to another commonly used classification, factor loadings of 0.70 or higher are considered to adequately explain the underlying construct, loadings of 0.50 or higher are regarded as practically meaningful, and 0.30 is accepted as the minimum acceptable threshold [5]. In our study, factor analysis revealed that factor loadings for the “*Oral Corticosteroid Phobia Scale*” ranged from 0.556 to 0.894, while factor loadings for the “*Intravenous Corticosteroid Phobia Scale*” ranged from 0.654 to 0.907. Based on these criteria, the observed factor loadings appear to fall within the acceptable to high range, suggesting that the underlying factor structures are adequately supported.

In our study, evaluation of model fit indices demonstrated that the χ²/df value was 1.87 for the oral form and 2.01 for the intravenous form; the SRMR values were 0.058 for the oral form and 0.056 for the intravenous form; the CFI values were 0.934 and 0.931, respectively; and the TLI values were 0.922 for the oral form and 0.920 for the intravenous form. A χ²/df ratio below 3 is indicative of very good to excellent model fit. For SRMR, values ≤ 0.05 reflect very good to excellent fit, whereas values between 0.05 and 0.08 indicate adequate to good fit. CFI values between 0.90 and 0.95 are considered to reflect adequate to good fit, while values ≥ 0.95 indicate very good to excellent fit. Regarding the TLI, values ≥ 0.80 are regarded as acceptable, values > 0.90 indicate good fit, and values ≥ 0.95 reflect excellent model fit [12, 14]. While these four fit indices indicated good to very good model fit, the RMSEA values were 0.084 for the oral form and 0.088 for the intravenous form, slightly exceeding the commonly accepted cutoff value of 0.08. The literature reflects ongoing debate regarding which fit indices should be prioritized and how many indices must meet predefined thresholds to conclude adequate model fit. In general, when the majority of fit indices demonstrate acceptable or good values, the overall model fit can be considered satisfactory [20]. In our study, given that the RMSEA values were close to the acceptable threshold and the other four fit indices demonstrated adequate fit, the overall model–data fit can be considered satisfactory. These scales provide an important tool for objectively assessing clinician attitudes toward corticosteroid use in pediatric rheumatology practice, identifying educational needs, and exploring the feasibility of steroid-sparing strategies in future research. Moreover, they establish a robust methodological foundation for further studies examining how clinician-based steroid fear influences treatment decision-making in pediatric rheumatic diseases.

### Strengths and limitations

The primary strength of this study lies in the development of one of the first instruments specifically designed to assess clinician attitudes toward systemic corticosteroid use in pediatric rheumatology. The content validity, construct validity, internal consistency, and temporal stability of both scales were comprehensively evaluated using modern psychometric methods. The multicenter design and the inclusion of 121 participants enhance the diversity of professional backgrounds and clinical experience represented in the sample. Although the number of pediatric rheumatology specialists worldwide is limited, the participation of clinicians from different centers contributes to the methodological robustness of the validation process.

Nevertheless, this study has several limitations. First, the inclusion of participants based on convenience sampling and voluntary participation may have introduced selection bias. Second, as the scales rely on self-reported clinician responses, the possibility of discrepancies between reported attitudes and actual clinical behavior cannot be excluded. Third, the cross-sectional design of the study precludes examination of the natural course of steroidophobia over time and limits the ability to assess its dynamic influence on clinical decision-making processes.

### Clinical and research implications

Importantly, the validation of a systemic corticosteroid phobia measure should not be interpreted as promoting avoidance of an essential medication. Systemic corticosteroids remain an important component of therapy in many pediatric rheumatologic conditions, particularly in scenarios requiring rapid disease control. Rather than encouraging therapeutic reluctance, this instrument offers a structured framework to better understand clinician beliefs and concerns that may influence treatment decisions. By making these perceptions explicit, the scale may help support reflective clinical practice and shared decision-making, and may contribute to more balanced discussions regarding steroid use, while preserving timely and effective disease control.

Beyond its conceptual contribution, the developed scales allow for the structured assessment of clinician attitudes in pediatric rheumatology practice, may help identify educational needs, and may clarify areas of uncertainty that arise in clinician–parent interactions. Moreover, they may serve as a basis for future research examining steroid-sparing strategies, standardized dosing approaches, and the potential impact of clinician perceptions on treatment outcomes.

## Conclusion

This study demonstrated that two newly developed instruments—the “*Oral Corticosteroid Phobia Scale*” and the “*Intravenous Corticosteroid Phobia Scale*”—are valid and reliable tools for assessing physician-based attitudes and concerns regarding systemic corticosteroid use in pediatric rheumatology practice. The high internal consistency coefficients, adequate item–total correlation values, and good factor loadings observed for both scales, together with strong correlations and high intraclass correlation coefficients (ICC) in test–retest analyses, support their psychometric soundness and temporal stability within the study sample.

The findings further suggest that pediatric rheumatology specialists experience substantial concern regarding adverse effects of both oral and intravenous corticosteroids, while the level of concern varies according to factors such as dosage, patient age, and clinical experience. Notably, lower corticosteroid phobia scores among clinicians with longer experience in pediatric rheumatology may indicate that clinical experience is associated with reduced reluctance toward corticosteroid use.

The developed scales may serve as useful tools in clinical research and practice, allowing structured evaluation of physician attitudes toward corticosteroid therapy, helping to identify potential educational needs, and informing discussions regarding steroid-sparing approaches. Nevertheless, further validation studies conducted in different countries, across various pediatric subspecialties, and in larger samples are warranted. Prospective studies exploring the relationship between clinician attitudes, patient/parent steroid fear, treatment adherence, and clinical outcomes are needed to better understand the broader implications of clinician-based steroid phobia.

## Supplementary Information

Below is the link to the electronic supplementary material.

Supplementary Material 1

## Data Availability

The datasets generated and/or analyzed during the current study are not publicly available due to participant confidentiality regulations but are available from the corresponding author upon reasonable request.
