# Supplementary material for: Systemic steroidophobia scale for pediatric rheumatology (SSS-PR): development and validation of a novel psychometric tool measuring clinician steroidophobia
Source: Pediatr Rheumatol Online J. 2026 Mar 26;24:31. doi: 10.1186/s12969-026-01200-z (PMC13141315; doi:10.1186/s12969-026-01200-z)
Supplement: Supplementary file 1 — Supplementary Material 1 [file 12969_2026_1200_MOESM1_ESM.docx]

| **Oral Corticosteroid Phobia Scale** | **n (%)** | | | | |
| --- | --- | --- | --- | --- | --- |
|  | **Strongly disagree** | **Disagree** | **Neutral** | **Partially agree** | **Strongly agree** |
| 1. I am concerned about the musculoskeletal side effects of oral corticosteroids. |  |  |  |  |  |
| 2. I am concerned about the neuropsychiatric side effects of oral corticosteroids. |  |  |  |  |  |
| 3. I am concerned about the metabolic–endocrinological side effects of oral corticosteroids. |  |  |  |  |  |
| 4. I am concerned about the ophthalmological side effects of oral corticosteroids. |  |  |  |  |  |
| 5. I am concerned about the immunological side effects of oral corticosteroids. |  |  |  |  |  |
| 6. I am concerned about the cardiovascular side effects of oral corticosteroids. |  |  |  |  |  |
| 7. I am concerned that oral corticosteroids may cause growth retardation in children. |  |  |  |  |  |
| 8. I am concerned about the dermatological side effects of oral corticosteroids. |  |  |  |  |  |
| 9. I am concerned about the hematological side effects of oral corticosteroids. |  |  |  |  |  |
| 10. I am concerned about the genitourinary side effects of oral corticosteroids. |  |  |  |  |  |
| 11. I am concerned about the gastrointestinal side effects of oral corticosteroids. |  |  |  |  |  |
| 12. I am reluctant to prescribe oral corticosteroids at doses exceeding 1 mg/kg/day. |  |  |  |  |  |
| 13. I am reluctant to prescribe oral corticosteroids to children younger than two years of age. |  |  |  |  |  |
| 14. I am reluctant to prescribe oral corticosteroids even at doses up to 1 mg/kg/day. |  |  |  |  |  |
| 15. Despite being aware of steroid-related side effects, I am reluctant to prescribe oral corticosteroids. |  |  |  |  |  |

| **Intravenous Corticosteroid Phobia Scale** | **n (%)** | | | | |
| --- | --- | --- | --- | --- | --- |
|  | **Strongly disagree** | **Disagree** | **Neutral** | **Partially agree** | **Strongly agree** |
| 1. I am concerned about the musculoskeletal side effects of intravenously administered corticosteroids. |  |  |  |  |  |
| 2. I am concerned about the neuropsychiatric side effects of intravenously administered corticosteroids. |  |  |  |  |  |
| 3. I am concerned about the ophthalmological side effects of intravenously administered corticosteroids. |  |  |  |  |  |
| 4. I am concerned about the cardiovascular side effects of intravenously administered corticosteroids. |  |  |  |  |  |
| 5. I am concerned that intravenously administered corticosteroids may cause growth retardation in children. |  |  |  |  |  |
| 6. I am concerned about the metabolic–endocrinological side effects of intravenously administered corticosteroids. |  |  |  |  |  |
| 7. I am concerned about the genitourinary side effects of intravenously administered corticosteroids. |  |  |  |  |  |
| 8. I am concerned about the hematological side effects of intravenously administered corticosteroids. |  |  |  |  |  |
| 9. I am concerned about the dermatological side effects of intravenously administered corticosteroids. |  |  |  |  |  |
| 10. I am concerned about the immunological side effects of intravenously administered corticosteroids. |  |  |  |  |  |
| 11. I am concerned about the gastrointestinal side effects of intravenously administered corticosteroids. |  |  |  |  |  |
| 12. I am reluctant to prescribe intravenous corticosteroids even at doses up to 2 mg/kg/day. |  |  |  |  |  |
| 13. I am reluctant to prescribe intravenous corticosteroids to children younger than two years of age. |  |  |  |  |  |
| 14. I am reluctant to prescribe intravenous corticosteroids at doses between 2 and 10 mg/kg/day. |  |  |  |  |  |
| 15. I am reluctant to prescribe intravenous corticosteroids at doses between 10 and 30 mg/kg/day. |  |  |  |  |  |
| 16. Despite being aware of steroid-related side effects, I am reluctant to prescribe corticosteroids via the intravenous route. |  |  |  |  |  |
